# Supplementary material for: Exploring spatio-temporal impact of COVID-19 on citywide taxi demand: A case study of New York City
Source: PLoS One. 2024 Apr 16;19(4):e0299093. doi: 10.1371/journal.pone.0299093 (PMC11020838; doi:10.1371/journal.pone.0299093)
Supplement: Attachment — Submitted filename: Response to Reviewers.docx [file pone.0299093.s001.docx]

Point-to-point responses to reviewers' comments

Reviewer #1：

In order to quantify the impacts of COVID-19 on city-wide taxi demand, the authors proposed series of spatio-temporal models to explore impact mechanism of pandemic on traffic demand pattern. Some findings are useful for policymakers and stakeholders. Here are some suggestions for improvement:

1.There are some words and phrases not used properly in this article, please examine the whole article and correct them, such as line 193 of the article, " at mid night " should be changed to " at midnight ". Line 508 of the article, "was build" should be changed to "was built".

Answer: Thank you very much for this suggestion. We have corrected some of the incorrect words and grammar.

2.This study focuses on New York City. Whether the result of this study is universal in other cities. This is worth discussing.

Answer: Thank you for highlighting the importance of discussing the generalizability of our study's findings beyond New York City. We have revised the Discussion section to acknowledge this limitation more explicitly. We now state that while our findings offer valuable insights into [specific aspects] of New York City, the extent to which these results can be generalized to other cities remains an open question. Additionally, we have included a comparison with relevant literature from studies conducted in other urban contexts. This comparison aims to provide a preliminary basis for understanding how our findings might align or differ in different settings. We have also suggested in the Conclusion section that further empirical studies are necessary to explore the applicability of our findings in diverse urban environments.

3.The Literature Review should be updated to the past two years. More recent articles should be cited.

Answer: Thanks for your suggestions. We have added [number] new references to our Literature Review, ensuring a comprehensive and up-to-date overview of the current research landscape.

4.The epidemic is over, the adaptation of policies and measures in post-epidemic period should be discussed.

Answer: Thank you for the insightful suggestion to discuss the adaptation of policies and measures in the post-epidemic period. We add to the conclusion by exploring the evolution of various policies and measures in response to the end of the epidemic, based on existing literature and case studies.

5.There is something wrong with the layout of the tables. The title should be placed above the table. Please examine the rest layout problems in the whole article.

Answer: Thank you for your suggestion. We have revised this issue.

Reviewer #2:

This study attempted to quantify the impacts of COVID-19 on city-wide taxi demand, and a time-space taxi demand model was built on NYC’s taxi and epidemic dataset. Some interesting conclusions were also drawn in this paper. Here are some suggestions for improvement:

1. The Literature Review in the article should be enriched. There have been lots of related studies in the past two years, and these literatures should be fully cited.

Answer: Thank you for your suggestion. We have added literature in recent years to enrich the content of the article.

2. There are too many figures in this article, and redundant images should be removed.

Answer: Thank you for your suggestion. We have deleted some unimportant images to make the article more concise.

3. There are some typographical problems, the title of the table should be placed above the table.

Answer: Thank you for your suggestion. We have revised this issue.

4. There are many grammatical errors in this article, please examine the whole article and correct them.

Answer: Thank you very much for this suggestion. We have corrected the grammatical errors.

5. There is something wrong with the syntax on line 120 of the article, " are illustrate " should be changed to " are illustrated".

Answer: Thank you very much for this suggestion. We have corrected the incorrect syntax.

6. The word in the text is misspelled in lines 190 and 191, “figure” should be “Figure”.

Answer: Thank you very much for this suggestion. We have corrected the incorrect words.

7. There is something wrong with the syntax on line 193 of the article, " at mid night " should be changed to " at midnight ".

Answer: Thank you very much for this suggestion. We have corrected the incorrect syntax.

8. There is something wrong with the syntax on line 508 of the article, "was build" should be changed to "was built".

Answer: Thank you very much for this suggestion. We have corrected the incorrect syntax.

9. The word in the text is misspelled, and in lines 528, “temporal-spatio” should be “spatio- temporal”.

Answer: Thank you very much for this suggestion. We have corrected the incorrect words.
